# MRI-based cerebellar volume measurements correlate with the International Cooperative Ataxia Rating Scale score in patients with spinocerebellar degeneration or multiple system atrophy

**DOI:** 10.1186/s40673-016-0052-4

**Published:** 2016-08-17

**Authors:** Daisuke Hara, Futaba Maki, Shigeaki Tanaka, Rie Sasaki, Yasuhiro Hasegawa

**Affiliations:** Department of Internal Medicine, Division of Neurology, St. Marianna University School of Medicine, 2-16-1 Sugao, Miyamae, Kawasaki, Kanagawa 216-8511 Japan

**Keywords:** MRI, Cerebellar volume, Spinocerebellar degeneration, Multiple system atrophy

## Abstract

**Background:**

Progression of clinical symptoms and cerebellar atrophy may vary among subtypes of spinocerebellar degeneration and multiple system atrophy. The aim of this cross-sectional study was to demonstrate the relationship between the International Cooperative Ataxia Rating Scale (ICARS) score and cerebellar volume derived from magnetic resonance imaging (MRI) in a broad spectrum of Japanese patients with cerebellar ataxia.

**Methods:**

A total of 86 patients with cerebellar ataxia (18 with cortical cerebellar atrophy, 34 with spinocerebellar ataxia, and 34 with multiple system atrophy) and 30 healthy subjects were studied. MRI-based cerebellar volume measurements were performed in all subjects using T1-weighted images acquired with a 1.5-T MRI scanner. The cerebellar volume/cranial anteroposterior (AP) diameter was used for statistical analysis.

**Results:**

Stepwise multiple regression analyses demonstrated that cerebellar volume/cranial AP diameter and midbrain AP/cranial AP diameter were significantly associated with the total score and domain I sub-score of ICARS. We found no interactions between these two anatomical factors in the ICARS total and domain I sub-scores. The main effects of these two predictors were statistically significant both in total and domain I sub-scores (*p* = 0.001 and 0.022, respectively).

**Conclusions:**

Cerebellar volume and midbrain AP diameter normalized to the cranial AP diameter were significantly correlated with the ICARS total and domain I sub-scores. Further longitudinal studies are warranted to explore the role of these MRI biomarkers for predicting disease progression.

## Background

Spinocerebellar degeneration (SCD) comprises a group of sporadic and hereditary neurodegenerative diseases with lesions involving the cerebellum and spinal cord. Many types of the dominantly inherited SCD have been named spinocerebellar ataxia (SCA), and natural histories of patients with the same genotype have been studied [1]. Multiple system atrophy (MSA), especially the cerebellar type, MSA-C, is another sporadic cerebellar ataxia with different clinical and pathological features from SCD. Clinical progression and prognosis are different among the various subtypes of degenerative cerebellar ataxia. Early accurate diagnosis and assessment of severity are essential for the best clinical management. Disease progression can be evaluated by using validated clinical rating scales such as the International Cooperative Ataxia Rating Scale (ICARS) [2], the Scale for Assessment and Rating of Ataxia (SARA) [3], or the unified MSA rating scale [4]. However, the large variation in rates of progression in different patients makes designing randomized, controlled studies for establishing a treatment for SCD and MSA difficult. Cerebellar volume measurement using magnetic resonance imaging (MRI) has been proposed as an imaging biomarker to predict and objectively evaluate differences in progression rates in these diseases [5–8]. Recently, the Alzheimer’s Disease Neuroimaging Initiative (ADNI) group demonstrated that MRI atrophy rate from baseline to 1 year is highly sensitive to the likelihood of clinical progression of dementia and calculated the sample size needed to detect a 25 % reduction in atrophy rate [9]. Similar utility of MRI-based cerebellar volume measurements in the degenerative cerebellar ataxia. The cross-sectional and longitudinal relationship between the cerebellar volume measurements obtained with MRI and the SARA score has been extensively studied in several types of SCA [5], however, such information with ICARS is scant [6].

A nationwide registry system of ‘intractable diseases’ including SCD and MSA has been established by the Ministry of Health, Labour, and Welfare in Japan [10, 11]. Since 2003, standardized assessment of all patients with SCD and MSA has been performed according to an inquiry sheet that includes five items from part I (Posture and Gait sub-component) and part II (Limb Ataxia sub-component) of ICARS. The aim of the present cross-sectional study was to evaluate the association between the ICARS score and MRI-based cerebellar volume in a broad spectrum of Japanese patients with cerebellar ataxia.

## Methods

### Patients

We studied 86 patients with SCD or MSA who were followed in our hospital from January 2004 to April 2013 (48 males; mean age 60.5 ± 10.7 years). To obtain normal values and establish the correction methods, 30 healthy volunteers from 18 to 83 years old were recruited as controls (15 males; mean age 64.1 ± 18.7 years). The patient group included 31 patients with MSA-C, three patients with MSA Parkinsonism-dominant subtype (MSA-P), 18 patients with cortical cerebellar atrophy (CCA), and 34 patients with SCA (13 with SCA6, seven with SCA3, three with SCA2, three with SCA1, two with SCA31, and six for whom the type of SCA was unknown). All patients were evaluated by MRI with standardized methods, and neurological examination using ICARS was performed within 45 days before or after MRI examination. ICARS is a validated 100-point ordinal scale (higher scores indicate greater ataxia) that quantifies ataxia in four categories of movement (I: posture and gait disturbances, II: kinetic (limb) functions, III: speech disorder, IV: oculomotor disorders).

Diagnosis of MSA was made in accordance with the second consensus statement including MRI findings [12]. Patients with a family history suggestive of dominant inheritance were diagnosed with SCA. After obtaining informed consent for genetic testing, patients were screened for SCA1, SCA2, SCA3, and SCA6. Further screening for SCA7, SCA8, SCA17, and SCA31 was performed in patients in whom the first screening was negative. When the other rare SCAs were suggested, further studies were performed according to the flow chart suggested by the Study Group on Ataxic Diseases, supported by the Ministry of Health, Labour and Welfare [11]. Patients with autosomal recessive cerebellar ataxia, such as Friedreich ataxia, ataxia-telangiectasia, ataxia with vitamin E deficiency etc., were excluded. CCA was defined as non-hereditary degenerative ataxia of unknown etiology [5, 12, 13]. The diagnosis of CCA was made based on the following criteria: progressive ataxia; disease onset after 20 years of age; no acute or subacute disease onset; informative and negative family history or no evidence of a causative gene mutation; no established symptomatic cause; and no possible or probable MSA [13]. This study was conducted in a single hospital, and the study protocol was approved by the St. Marianna University Bioethics Committee and written informed consent was obtained by normal volunteers. Written informed consent from patient was waived because of the retrospective analysis of anonymized data.

### Measurements of brain structures

Cranial MRI was performed using a 1.5-T scanner (Philips Electronics Japan, Tokyo, Japan). Brain volume measurement software (TRI/3D-VOL; Ratoc System Engineering, Tokyo, Japan) [14–16] was used to measure brain structures and volumes using Digital Imaging and Communications in Medicine (DICOM) data from the T1-weighted sagittal images (TE, 15 ms; TR, 520 ms; flip angle, 90°; number of excitations, 3; slice thickness, 4 mm; matrix, 272 × 256 sagittal sections).

**Fig. 1:**
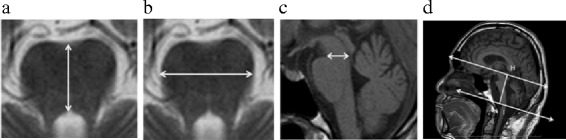
Measurements of infratentorial structures. Anteroposterior diameter of the pons was measured at the level of the opening of the sella turcica (**a**). Transverse diameter of the pons was measured as the distance between two points perpendicular to the midline along the tangent lateral to the pons (**b**). Midbrain anteroposterior diameter was measured at a midline level on a sagittal section (**c**). Cerebellar height was defined as the distance between the apex of the anterior lobe of the cerebellum and the apex of the cerebellar flocculus (H-I line was perpendicular to the AC-PC line) (**d**)

**Fig. 2:**
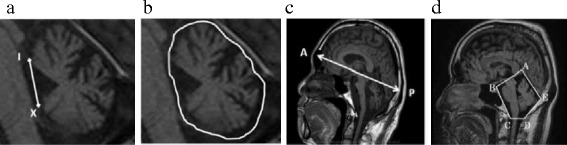
Segmentation of the cerebellum on MR images. The cerebellum was defined as the area lateral to the line connecting the anterior lobe of the cerebellum (I segment) and cerebellar flocculus (X segment) (**a**). Using DICOM data from cranial MRI T1 sagittal sections, the cerebellum was traced freehand on each cerebellum MRI sagittal section slice (**b**). Then, on the central slice, the fourth ventricle and surrounding cerebellar tissue were selected, and the cerebellum was automatically extracted. The cerebellar volume was determined by linear interpolation from the automatically extracted cerebellar tissue area and slice thickness. The anteroposterior diameter of the cranium (the distance between two points, A and P, at which the skull and the anterior commissure-posterior commissure line intersect) was used for the cerebellar volume correction (**c**). The posterior cranial fossa volume was determined by five anatomical landmarks (A: the upper tentorial end, B: infundibular recess, C: basilar impression, D: posterior pole of the hard palate, E: internal occipital protuberance) and used for additional cerebellar volume correction (**d**)

In all patients and control subjects, pons anteroposterior (AP) diameter, pons transverse diameter, midbrain tegmentum diameter, cerebellar height, cranial AP diameter, cerebellar volume, and posterior cranial fossa volume were measured. Pons AP diameter was measured in the pons midline at the level of the opening of the sella turcica. The transverse diameter of the pons was measured as the distance between two points perpendicular to the midline along the tangent lateral to the pons. The diameter of the midbrain tegmentum was measured at the midline level on a sagittal section. Cerebellar height was defined as the distance, including the cerebellum, drawn parallel to the cranial AP diameter (Fig. 1). Cranial AP diameter, which was defined as the distance between two points at which the skull and the anterior commissure – posterior commissure (AC-PC) line intersected, was measured (Fig. 2).

### Cerebellar volume measurement and correction for head size

Segmentation of the cerebellum on MR images is challenging. No clear anatomical landmarks exist on cranial MRI to distinguish the cerebellum, cerebellar peduncles, and brainstem. In this study, for the boundary between the cerebellum and brainstem on each sagittal section slice, the cerebellum was defined as the area lateral to the line connecting the anterior lobe of the cerebellum (I segment) and cerebellar flocculus (X segment) (Fig. 2a). Using DICOM data from cranial MRI T1 sagittal sections, the cerebellum was traced freehand on each cerebellum MRI sagittal section slice (Fig. 2b). Then, on the central slice, the fourth ventricle and surrounding cerebellar tissue were selected, and the cerebellum was automatically extracted using discriminant analysis. Briefly, a histogram of the image pixel numbers was created, and then a threshold was determined for the maximum ratio of the between-class to within-class variance (resolution) of the pixel numbers in the regions to be separated by binarization. Using this method, the ventricle around the cerebellum, with a pixel number different than the cerebellar tissue, was separated from the cerebellum that was traced freehand, and thus, only the cerebellum was automatically extracted. The same procedure was performed for all slices. Cerebellar volume was determined by linear interpolation from the automatically extracted cerebellar tissue area and slice thickness.

Inter-rater variability and test-retest reliability for the MRI-based cerebellar volume measurements were calculated by using 12 randomly extracted patients from our data set. Cerebellar volume was measured three times by three experienced neurologists (DH, FM and ST) who were blinded to the patient clinical information. The intraclass correlation coefficients were 0.988 for inter-rater variability and 0.994 for test-retest reliability.

For statistical analysis, the cerebellar volume of all subjects was corrected for individual head size differences based on two different methods: the AP diameter of the cranium (the distance between two points at which the skull and the AC-PC line intersect) or the posterior cranial fossa volume. We calculated the cerebellar volume/cranial AP diameter as the volume index and also calculated the cerebellar volume/posterior cranial fossa volume. The posterior fossa was defined in accordance with methods proposed by Urbizu et al. [17] and Smoker et al. [18] (Fig. 2), and its volume was measured with brain measurement software.

### Statistical analyses

The characteristics of patients are given as the mean and standard deviation unless otherwise indicated. Unpaired Student’s *t*-tests or a Mann–Whitney test were used to compare two groups, and χ^2^ tests were used for nominal parameters. Pearson’s or Spearman’s correlation coefficients were used to test the strength of association between two variables. Comparisons among three subgroups were made using ANOVA or a Kruskal-Wallis test. Multiple linear regression analysis was used to investigate factors significantly associated with cerebellar volume in healthy subjects. Stepwise multiple linear regression analyses were used to determine factors significantly associated with the ICARS total or subdomain scores. Then these factors were grouped into tertiles. General linear models were used to test the main and interaction effects of these categorical variables. The Tukey honestly significant difference test was used for the post-hoc comparison of variables. Values of *p* < 0.05 were considered significant. All statistical analyses were performed using SPSS version 21 (IBM SPSS Statistics for Windows, IBM Corp., Armonk, NY).

## Results

In healthy subjects, the midbrain AP diameter and pons AP diameter were significantly correlated with cranial AP diameter (*r* = 0.386 and *r* = 0.489). The cerebellar volume was significantly correlated with body height, cranial AP diameter, and posterior cranial fossa volume (*r* = 0.700, *r* = 0.611, and *r* = 0.459, respectively, Table 1). Multiple linear regression analysis demonstrated that the cranial AP diameter was significantly associated with cerebellar volume when the following variables were forced into the model simultaneously: age, sex, body height, body weight, cranial AP diameter and posterior cranial fossa volume (*p* = 0.042, Table 2). For further statistical analyses, we used the cranial AP diameter to correct the head size of each patient.

**Table 1:** Correlation matrix of age, body height, body weight, cranial AP diameter, and posterior cranial fossa volume with MRI measurements of infratentorial structures in healthy control subjects

	Age (years)	Body height (cm)	Body weight (kg)	Cranial AP diameter (mm)	Posterior cranial fossa volume (ml)
Midbrain AP diameter (mm)	0.022	0.228	0.016	0.386^*^	0.315
Pons AP diameter (mm)	0.290	0.269	0.305	0.489^**^	0.114
Pons transverse diameter (mm)	−0.134	−0.037	−0.073	0.195	0.215
Cerebellar height (mm)	0.100	0.116	0.093	0.189	0.166
Cerebellar volume (ml)	−0.353	0.700^**^	−0.321	0.611^**^	0.459^*^

Values are Pearson’s correlation coefficients

*p < 0.05, **p < 0.001 (two sided)

**Table 2:** Multiple linear regression analysis of factors significantly associated with cerebellar volume in healthy control subjects

	B	95 % confident interval	*p* value
Male sex	3.215	−11.831 to 18.260	0.650
Age (years)	−0.066	−1.375 to 1.242	0.914
Body height (cm)	0.886	−0.502 to 2.274	0.190
Body weight (kg)	0.023	−1.165 to 1.211	0.966
Cranial AP diameter (mm)	0.655	0.027 to 1.283	0.042
Posterior cranial fossa volume (ml)	0.060	−0.150 to 0.270	0.543

Patient’s characteristics are shown in Table 3. The posterior cranial fossa volume, pons AP diameter, pons transverse diameter, cerebellar height, and cerebellar volume in patients with cerebellar ataxia were significantly smaller than those in healthy subjects. The pons AP diameter, pons transverse diameter, cerebellar height, and cerebellar volume in patients were also smaller than those in healthy subjects, even after correction by the cranial AP diameter. Age, midbrain AP diameter, pons transverse diameter, disease duration, and ICARS domain I sub-score were significantly different among the subgroup of patients with cerebellar ataxia (*p* = 0.027, *p* = 0.017, *p* = 0.001, *p* = 0.001, *p* = 0.001, respectively). The midbrain AP diameter and pons transverse diameter were also significantly different after correction by cranial AP diameter. Post-hoc analysis demonstrated that patients with SCA were younger than those with CCA (*p* < 0.05), the midbrain AP diameter in patients with MSA was smaller than in those with CCA (*p* < 0.015), and the pons transverse diameter in patients with MSA was significantly smaller than in those with CCA (*p* < 0.01) and SCA (*p* < 0.01). Disease duration in patients with MSA was significantly shorter than that in those with SCA (*p* < 0.001). The ICARS domain I sub-score in MSA was significantly higher than in those with SCA (*p* = 0.047) and CCA (*p* = 0.001).

**Table 3:** Patient’s characteristics and measurements of infratentorial structures

	Healthy control subjects	All patients			Subtype		
	(*n* = 30)	(*n* = 86)	*p* value^b^	SCA (*n* = 34)	CCA (*n* = 18)	MSA (*n* = 34)	*p* value^c^
Age (years)	64.2 ± 18.7	60.5 ± 10.7	0.001	57.0 ± 12.0	65.2 ± 10.7	61.3 ± 8.1	0.027
Male sex	15 (50 %)	48 (55.8 %)	0.526	19 (55.9 %)	7 (38.9 %)	22 (64.7 %)	0.284
Body height (cm)	163.5 ± 6.7	160.4 ± 8.2	0.285	161.1 ± 8.6	156.9 ± 7.9	161.5 ± 7.7	0.124
Body weight (kg)	64.2 ± 18.7	57.3 ± 10.7	0.039	57.1 ± 10.8	57.9 ± 11.7	57.2 ± 10.3	0.959
MRI measurement							
Cranial AP diameter (mm)	181.6 ± 10.8	184.7 ± 9.3	0.074	184.1 ± 9.3	181.2 ± 11.7	187.1 ± 7.2	0.086
Posterior cranial fossa volume (ml)	274.0 ± 26.7	260.0 ± 30.0	0.815	264.7 ± 33.8	252.6 ± 20.2	259.3 ± 29.1	0.377
Midbrain AP diameter (mm)	15.1 ± 1.9	15.0 ± 2.0	0.711	14.9 ± 1.9	16.1 ± 1.9	14.5 ± 1.9	0.017
Pons AP diameter (mm)	22.77 ± 1.5	14.9 ± 2.0	0.711	15.1 ± 1.8	14.6 ± 1.9	15.1 ± 2.3	0.723
Pons transverse diameter (mm)	28.4 ± 4.3	26.4 ± 3.4	0.394	26.9 ± 2.5	29.4 ± 3.1	24.4 ± 3.0	0.001
Cerebellar height (mm)	55.5 ± 3.3	47.1 ± 6.1	0.000	48.4 ± 50	47.8 ± 6.4	45.6 ± 6.7	0.152
Cerebellar volume (ml)	117.6 ± 13.7	82.7 ± 16.6	0.192	83.4 ± 16.6	84.9 ± 16.2	78.8 ± 16.5	0.217
Correction for cranial size^a^							
Corrected Midbrain AP diameter	0.083 ± 0.010	0.081 ± 0.011	0.417	0.081 ± 0.010	0.089 ± 0.011	0.078 ± 0.011	0.002
Corrected Pons AP diameter	0.126 ± 0.008	0.083 ± 0.011	0.417	0.082 ± 0.010	0.081 ± 0.013	0.081 ± 0.011	0.928
Corrected Pons transverse diameter	0.157 ± 0.024	0.144 ± 0.024	0.406	0.146 ± 0.015	0.163 ± 0.020	0.131 ± 0.018	0.001
Corrected Cerebellar height diameter	0.306 ± 0.023	0.256 ± 0.038	0.005	0.264 ± 0.030	0.265 ± 0.042	0.245 ± 0.040	0.061
Corrected Cerebellar volume diameter	0.65 ± 0.06	0.49 ± 0.09	0.011	0.46 ± 0.09	0.47 ± 0.09	0.42 ± 0.09	0.100
Disease duration (years)	---	7.6 ± 6.4		10.5 ± 8.0	8.2 ± 4.6	4.1 ± 2.7	0.001
ICARS score							
Total score	---	36.7 ± 15.3		35.7 ± 12.1	31.4 ± 11.3	40.5 ± 19.0	0.206
Domain I	---	15.0 ± 7.3		13.7 ± 6.4	10.7 ± 5.5	18.5 ± 7.6	0.001
Domain II	---	16.5 ± 8.5		16.4 ± 6.3	15.4 ± 6.6	17.11 ± 1.1	0.800
Domain III	---	3.21 ± 0.5		2.9 ± 1.1	3.2 ± 1.7	3.4 ± 1.7	0.468
Domain IV	---	1.81 ± 0.3		1.9 ± 1.4	1.8 ± 1.3	1.6 ± 1.3	0.263

SCA indicates spinocerebellar ataxia; CCA, cortical cerebellar atrophy; MSA, multiple system atrophy

^a^ each factor was divided by the cranial AP diameter

^b^ All patients vs. healthy control subjects (unpaired Student’s *t*-test and *χ*2 test)

^c^ one-way analysis of variance for the continuous variables and Kruskal-Wallis test for the ICARS score. *χ*2 test for the male sex distribution

Stepwise multiple linear regression analyses were performed with total ICARS scores and scores of each domain (Table 4). In these analyses, age, sex, midbrain AP/cranial AP diameter, pons AP/cranial AP diameter, pons transverse/cranial AP diameter, cerebellar height/cranial AP diameter, and cerebellar volume/cranial AP diameter were used as independent variables. The cerebellar volume/cranial AP diameter and midbrain AP/cranial AP diameter were determined to be significantly associated factors with the total score and domain I sub-score. The domain II and domain III scores were determined to be significantly associated factors with the cerebellar volume/cranial AP diameter and midbrain AP/cranial AP diameter, respectively. For domain IV, no factor was selected as an significantly associated factor (Table 4).

**Table 4:** Stepwise multiple linear regression analyses of factors significantly associated with the ICARS score

Predictive factors for the ICARS score	B	SD	*p* value
Total score			
Cerebellar volume/cranial AP diameter	−60.284	16.366	0.000
Midbrain AP/cranial AP diameter	−315.395	132.638	0.020
Domain I sub-score			
Cerebellar volume/cranial AP diameter	−32.001	7.367	0.000
Midbrain AP/cranial AP diameter	−200.440	59.702	0.001
Domain II sub-score			
Cerebellar volume/cranial AP diameter	−28.327	9.480	0.004
Domain III sub-score			
Midbrain AP/cranial AP diameter	−31.384	13.837	0.026
Domain IV sub-score			
No factor was selected			

Stepwise multiple linear regression analyses were performed separately for the total score and each sub-score in the four domains. Age, sex, midbrain AP/cranial AP diameter, pons AP/cranial AP diameter, pons transverse/cranial AP diameter, cerebellar height/cranial AP diameter, and cerebellar volume/cranial AP diameter were used as dependent variables

*AP* anteroposterior, *SD* standard deviation

**Fig. 3:**
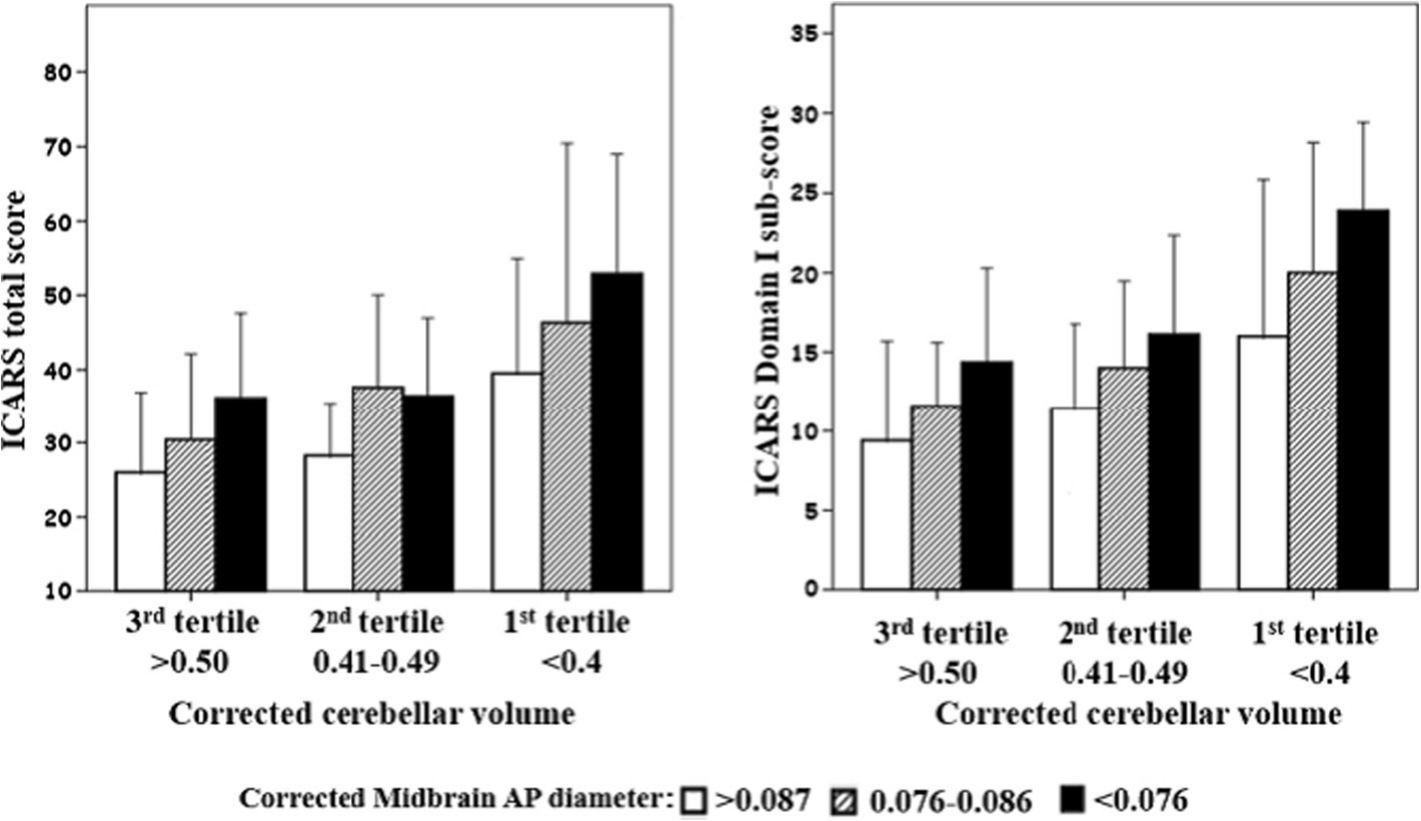
ICARS total score and domain I sub-score in relation to two significantly associated factors. For the ICARS total score and domain I sub-score, we found no interaction between the corrected cerebellar volume and the corrected midbrain AP diameter (F[2,77] = 0.259, *p* = 0.903 and F[2,77] = 0.161, *p* = 0.958, respectively). For the ICARS total score, the main effects of these two factors were significant (F(2,77) = 8.635, *p* = 0.001 and F(2,77) = 4.017, *p* = 0.022, respectively). For the domain I sub-score, the main effects of these two factors were also significant (F(2,77) = 5.757, *p* = 0.005 and F(2,77) = 11.265, *p* = 0.001, respectively)

Figure 3 shows ICARS total and domain I scores in relation to tertiles of midbrain AP/cranial AP diameter and cerebellar volume/cranial AP diameter. We found no interactions between the two factors in ICARS total and domain I scores (F[2,77] = 0.259, *p* = 0.903 and F[2,77] = 0.161, *p* = 0.958, respectively). For the total score, the main effects of these two factors were significant (F(2,77) = 8.635, *p* = 0.001 and F(2,77) = 4.017, *p* = 0.022, respectively). Post-hoc analysis showed that the 1st tertile vs. the 2nd tertile and the 1st tertile vs. the 3rd tertile of the midbrain AP diameter/cranial AP diameter were significantly different (*p* = 0.001 and 0.022, respectively). Also, the 1st tertile vs. the 3rd tertile of the cerebellar volume/cranial AP diameter was significantly different (*p* = 0.001). For the domain I sub-score, the main effects of these two factors (midbrain AP/cranial AP diameter and cerebellar volume/cranial AP diameter) were also significant (F(2,77) = 5.757, *p* = 0.005 and F(2,77) = 11.265, *p* = 0.001, respectively). Post-hoc analysis demonstrated that the 1st tertile vs. the 3rd tertile and the 2nd tertile vs. the 3rd tertile of the midbrain AP diameter/cranial AP diameter were significantly different (*p* = 0.006 and 0.048, respectively). Also, the 1st tertile vs. the 2nd tertile and the 1st tertile vs. the 3rd tertile of the cerebellar volume/cranial AP diameter were significantly different (*p* = 0.002 and 0.001, respectively).

**Fig. 4:**
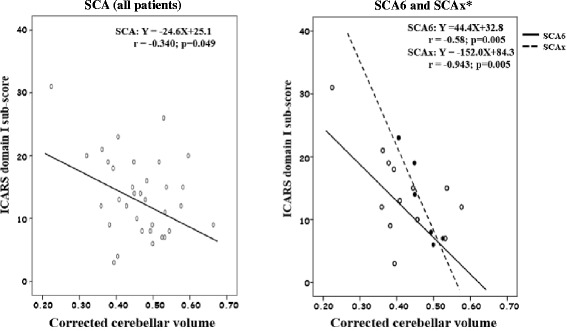
Correlation between the corrected cerebellar volume and the ICARS domain I sub-score in patients with spinocerebellar ataxia. * SCAx indicates that the type of SCA was unknown

**Fig. 5:**
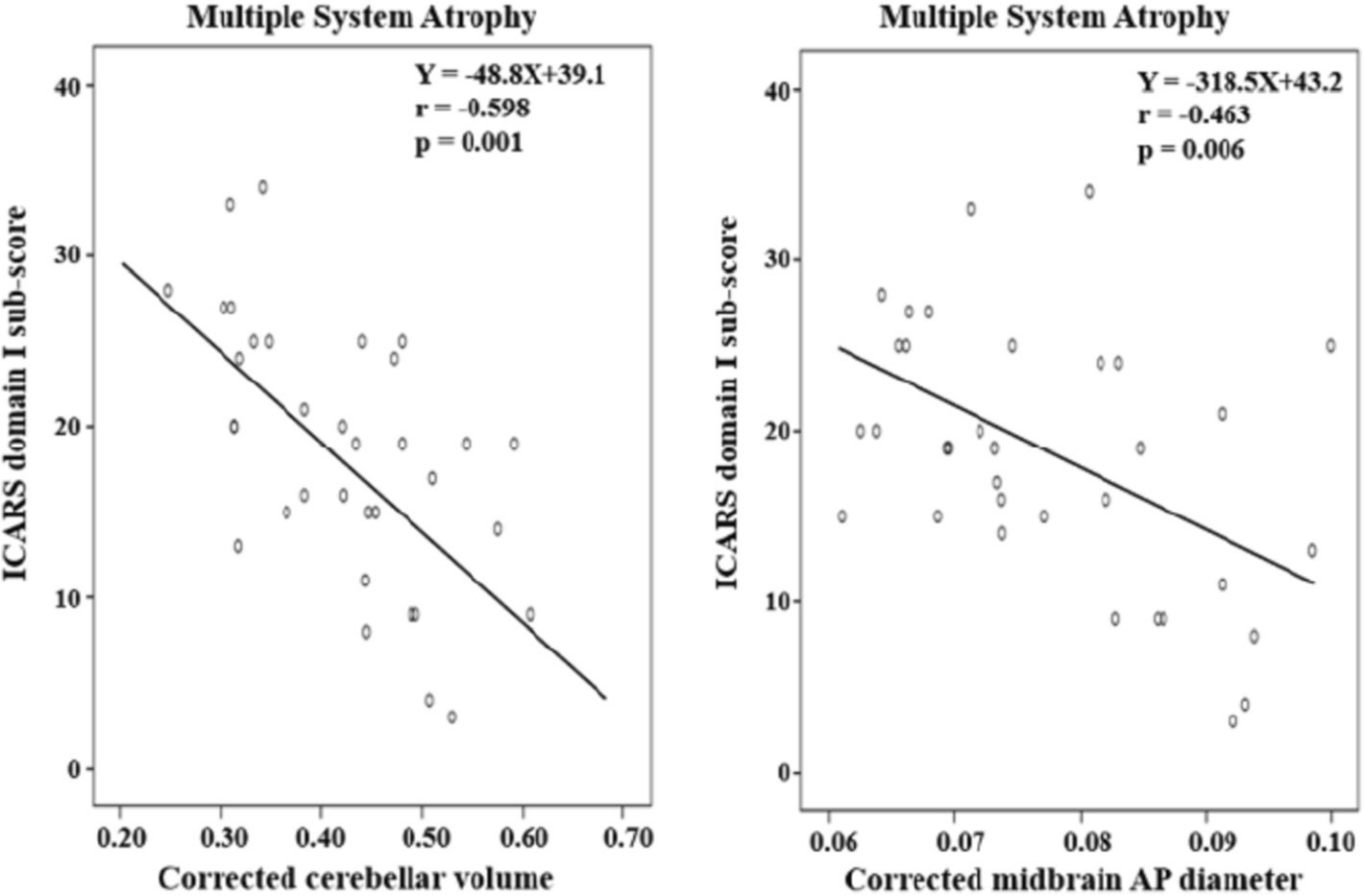
Correlation between MRI biomarkers and the ICARS domain I sub-score in patients with multiple system atrophy

Correlation coefficients between ICARS scores and two MRI biomarkers according to subtypes of cerebellar ataxia are shown in Table 5. The numbers of patients with SCA1, SCA2, and SCA31 were too small to calculate a correlation coefficient. In patients with SCA6, the unknown type of SCA, and MSA, a significant correlation was demonstrated between the ICARS total score and the corrected cerebellar volume. Also, a significant correlation between the domain I sub-score and the corrected cerebellar volume was observed in all SCA patients, SCA6 patients, those in whom the type of SCA was unknown, and those with MSA. In patients with MSA, the corrected midbrain AP diameter was also correlated with the ICARS domain I sub-score. Scattergrams are shown in Figs. 4 and 5.

**Table 5:** Correlation coefficients between ICARS scores and two MRI biomarkers according to the subtypes of cerebellar ataxia

				ICARS total score				ICARS domain I		
Subtype		n	Cerebellar volume	*p* value	Midbrain AP diameter	*p* value	Cerebellar volume	*p* value	Midbrain AP diameter	*p* value
SCA	all patients	34	−0.306	0.079	−0.176	0.319	−0.340	0.049	−0.168	0.342
	SCA1	3	----	----	----	----	----	----	----	----
	SCA2	3	----	----	----	----	----	----	----	----
	SCA3	7	−0.068	0.885	−0.279	0.545	−0.094	0.842	−0.395	0.380
	SCA31	2	----	----	----	----	----	----	----	----
	SCA6	13	–0.571*	0.042	−0.251	0.409	–0.580*	0.038	−0.311	0.301
	SCAx	6	–0.943*	0.005	0.029	0.957	–0.943*	0.005	0.371	0.468
CCA	all patients	18	0.155	0.539	0.023	0.928	−0.073	0.772	−0.050	0.843
MSA	all patients	34	–0.593*	0.001	−0.367^*^	0.033	–0.598*	0.001	–0.463*	0.006

Values of correlation coefficient are Spearman’s rho

*SCA* indicates spinocerebellar ataxia, *SCAx* the type of SCA was unknown, *CCA* cortical cerebellar atrophy, *MSA* multiple system atrophy

* *p* < 0.05

## Discussion

The cerebellar volume in our control group was 117.6 ± 13.7 ml, which is slightly less than that reported by Klockgether et al. (132.7 ± 15.8 ml) [6] and by the Rotterdam Study (men, 129.7 ± 0.25 ml; women, 126.2 ± 0.22 ml) [19, 20]. However, considering the smaller body habitus of Japanese persons, our study value seems reasonable. Eichler et al. [7] reported cerebellar volumes of 132.6 ± 11.3 cm^3^ in normal subjects, 119.0 ± 17.2 cm^3^ in SCA3 patients, and 98.1 ± 14 cm^3^ in SCA6 patients. A significantly smaller pons AP diameter in SCA6 patients (2.23 ± 0.17 cm) than in normal subjects (2.37 ± 0.26 cm) has also been reported [21]. Our study also found a smaller pons AP diameter, pons transverse diameter, cerebellar height, and overall cerebellar volume in SCD patients compared to healthy subjects, which is in agreement with previously reported findings [7, 21].

The size and volume of each brain structure vary with an individual’s stature, age, or sex [22]. For clinical application of MRI measurements of intracranial structures, correction for these individual differences is necessary. However, no correction methods have been established to date. Instead, various imaging and correction techniques have been used depending on each study’s objective [6–8, 23–25]. By calculating the cerebellar volume/cranial AP diameter in our study, age- and sex-related differences in healthy subjects were eliminated. Therefore, this correction method was used to examine the correlation between MRI-based measurements of intracranial structures with ICARS total and domain scores in all SCD patients and in each subtype.

Only in MSA were there correlations with both cerebellum and midbrain, in SCA there were only correlations with cerebellum, and in CCA there were no significant associations (Table 5). These findings may represent the different pathology among these subtypes. In future therapeutic trials, it is desirable to examine the efficacy of specific therapy for the degenerative cerebellar ataxia in its early stage. However, the diagnosis of vast majority of patients may not confirmed in the early stage except genetically diagnosed patients [26]. Our study demonstrated that the corrected cerebellar volume and midbrain size were significantly correlated with the total score and domain I sub-score of ICARS in a broad range of subtypes of degenerative cerebellar ataxia (Table 4 and Fig. 3). Moreover, no interaction between these two MRI biomarkers was demonstrated, and the main effects of these markers were significant. The correlation of MRI biomarkers with the domain I sub-score was much better than that with the total score. This result coincides with a recent systematic review that demonstrated that the domain I sub-score (Posture and Gait sub-component) of ICARS has the most robust psychometric property and acceptable clinical utility among 16 rating scales for ataxia [26].

MRI-based cerebellar volume measurement still remains challenging, due to thin sulci and gyri and close connection to the brainstem. However, significant correlation between cerebellar volume and clinical severity have been confirmed by several sophisticated MRI methods in different forms of inherited ataxia [25, 27–30]. In this study, we analyzed the DICOM data obtained by routine MRI studies in order to utilize our results in a future therapeutic trial. In the present cross-sectional study, we evaluated the association between the ICARS score and MRI-based cerebellar volume in a broad spectrum of Japanese patients with cerebellar ataxia. Several authors criticized that the redundant and overlapping nature of several items of ICARS gave rise to a considerable number of contradictory ratings [31, 32]. However, it is reasonable to assume that ICARS and SARA are quite convergent and further validation in a range of clinical settings should be continued.

In our study of each subtype of degenerative cerebellar ataxia, no significant correlation was found between the cerebellar volume and midbrain size in SCA3. Pathology in SCA3 is characterized by cerebellar cortical degeneration and brainstem atrophy [33], and in addition to cerebellar ataxia, symptoms can involve multiple systems such as the extrapyramidal tract. Eichler et al. [7] reported a correlation between ICARS and both the cerebellar and brainstem volumes in SCA6, but only with brainstem volume in SCA3. Thus, the progression of atrophy in SCA3 may differ from that in other SCA subtypes.

The European Integrated Project on Spinocerebellar Ataxias (EUROSCA) registry has demonstrated genotype-specific patterns of atrophy progression in SCA1, SCA3, and SCA6 with quantitative volumetry and voxel-based morphometry imaging. This study also showed that MRI measurements are highly sensitive for detecting changes and are superior to the SARA scale [5]. In addition, the repeat length of the expanded allele showed a weak negative correlation with atrophy in SCA3 and SCA1, whereas no correlation was found with SCA6 [34].

CCA, which is nearly synonymous with sporadic adult-onset ataxia of unknown origin [35] and idiopathic cerebellar ataxia [36] in Western countries, presents as a pure cerebellar ataxia [11]. Although CCA mainly presents with cerebellar symptoms, in our study, contrary to expectation, we found no correlation between the ICARS score and cerebellar volume. As evident from the diagnostic criteria for CCA, the most likely reason is that various disorders of unknown cause with different genotypes usually lead to this diagnosis. However, the prevalence of CCA in Japan has reached about 9000, and the development of treatment for these patients is important. The significance of MRI biomarkers requires further investigation in a longitudinal study.

MSA subtypes differ by country. MSA-C accounts for ≥60 % of cases in Japan [10, 37], which is similar to other Asian countries [38, 39]. In contrast, MSA-P accounts for ≥60 % of cases in Western countries. The pathology in MSA patients also clearly shows ethnic differences [40]. The correlation between MRI measurements and ICARS scores found in this study may be promising for use in the clinical evaluation of Japanese MSA patients.

This study has some limitations. First, MRI-measured values were corrected only by AP diameter, but we did not perform age correction. Cerebellar atrophy associated with aging has seldom been reported [8, 20], but in a study by Schulz et al., cerebellar volume decreases with age, and this decrease is more prominent in men than in women [8]. However, in the healthy control group in our study (mean age: 64.1 ± 18.7 years), no correlation was observed between age and various MRI measurements. Therefore, age correction was not performed. Nevertheless, some age correction may be necessary in future long-term longitudinal studies. Second, because of the small number of patients, the correlation between MRI measurements and ICARS scores could not be clarified for some SCA genotypes and we could not analyze the data for MSA-C and MSA-P separately. However, the ICARS domain I sub-score is evaluated in many patients in Japan. Thus, by using the simple MRI measurement techniques described in this paper, acquisition of data from a larger number of patients may be possible.

## Conclusions

The cerebellar volume and midbrain AP diameter normalized to the cranial AP diameter were significantly correlated with the ICARS total score and domain I sub-score. To the best of our knowledge, this is the first study to elucidate the relationship between the ICARS score and MRI biomarkers in Japanese patients with degenerative cerebellar ataxia. Further longitudinal studies are warranted to explore the role of these MRI biomarkers for prediction of disease progression.

## Abbreviations

AC-PC line: Anterior Commissure - Posterior Commissure Line
AP: Anteroposterior
CCA: Cortical cerebellar atrophy
DICOM: Digital imaging and communications in medicine
ICARS: International cooperative ataxia rating scale score
MRI: Magnetic resonance imaging
MSA: Multiple system atrophy
MSA-C: Multiple system atrophy, especially the cerebellar type
MSA-P: Multiple System Atrophy, Especially The Parkisonism Type, Sca, Spinocerebellar Ataxia
SARA: Scale for the assessment and rating of ataxia
SCD: Spinocerebellar degeneration
